# Supplementary material for: Dynamic Changes in Drinking Behaviour among Subpopulations of Youth during the COVID-19 Pandemic: A Prospective Cohort Study
Source: Healthcare (Basel). 2023 Jul 5;11(13):1945. doi: 10.3390/healthcare11131945 (PMC10340784; doi:10.3390/healthcare11131945)
Supplement: Supplementary file 1 [file healthcare-11-01945-s001.zip › healthcare-2457440-supplementary.pdf]

Supplementary Tables

Supplementary Table S1. Comparison of characteristics of linked and non-linked data from students attending the 81 linked-longitudinal COMPASS secondary schools across the three study waves (2018/19, 2019/20, and 2020/21).

	Linked (n= 5368)	Non-linked (n=42286)	P (χ^2/t-test)
Sex (female)	60.2 (3211)	48.1% (20,065)	<0.001
Age			<0.001
12-13	36.9 (1974)	19.36% (7,841)	
14	36.3 (1944)	30.2 (12312)	
15-18	26.7 (1427)	50.5 (20559)	
Race/ethnicity			<0.001
White	79.9 (4288)	70.6 (29843)	
Black	1.8 (95)	4.2 (1768)	
Asian	6.2 (332)	7.7 (3256)	
Latin/Hispanic	1.0 (54)	2.4 (1015)	
Mixed/Others	11.2 (599)	15.1 (6404)	
Drinking level			<0.001
None	66.5 (3539)	55.1 (22916)	
Occasional	16.8 (896)	18.5 (7666)	
Monthly	13.9 (740	19.2 (7997)	
Weekly	2.8 (149)	7.2 (2981)	
Binge drinking			<0.001
None	84.2 (4516)	73.1 (30824)	
Occasional	9.4 (506)	13.1 (559)	

Monthly	5.7 (303)	10.8 (4532)
Weekly	0.7 (35)	3.0 (1258)

Supplementary Table S2. Latent transition model fit indices

	-Log likelihood	G^2	df	AIC	Adjusted BIC
Overall					
2-class	24707.4	5649.0	4078	5683.0	5794.9
3-class	23186.3	2606.9	4063	2670.9	2881.7
4-class	22755.7	1745.6	4044	1847.6	2183.6
5-class	22578.8	1381.8	4021	1529.8	2017.3
6-class	22498.8	1231.9	3994	1433.9	2099.3
FEMALES					
2-class	15113.7	3564.4	4078	3598.4	3701.7
3-class	14218.1	1773.0	4063	1836.9	2031.5
4-class	14032.1	1401.0	4044	1503.0	1813.0
5-class	13860.1	1057	4021	1205	1654.9
6-class	13816.2	969.3	3994	1171.3	1785.3
MALES					
2-class	9488.2	2715.3	4078	2749.3	2845.6
3-class	8845.2	1429.4	4063	1493.4	1674.7
4-class	8670.4	1079.7	4044	1181.6	1470.7
5-class	8600.3	939.5	4021	1087.5	1506.9
6-class	8558.4	855.8	3994	1057.8	1630.3

G^2: Likelihood ratio statistics; df: degrees of freedom; AIC: Akaike Information Criteria; BIC: Bayesian Information Criteria.

Supplementary Table S3. Comparison of transition probabilities between latent statuses of youth alcohol consumption among two cohorts during one year before the COVID-19 pandemic and two years during the pandemic in females and males attending the 81 linked-longitudinal COMPASS schools across the three study waves (2018/19, 2019/20, and 2020/21)

	Abstainer		Occasional drinker-no binging		Occasional binge drinker		Monthly binge drinker		Weekly binge drinker	
Cohort	T2a	T2b	T2a	T2b	T2a	T2b	T2a	T2b	T2a	T2b
**Females**										
**Pre-pandemic (T1)**										
Abstainer	**0.66**	**0.69**	0.22	0.22	0.07	0.03	.05	0.04	0.00	.01
Occasional drinker-no binging	0.01	0.01	**0.35**	**0.52**	0.41	0.25	0.22	0.21	0.00	0.02
Occasional binge drinker	0.00	0.01	0.14	0.23	**0.45**	**0.32**	0.37	0.37	0.03	0.05
Monthly binge drinker	0.00	0.04	0.05	0.13	0.06	0.02	**0.71**	**0.64**	0.17	0.17
Weekly binge drinker	0.00	0.01	.00	0.00	0.20	0.00	0.01	0.13	**0.80**	**0.86**
**Early- pandemic (T2)**										
Abstainer	**0.73**	**0.78**	0.17	0.18	0.06	0.02	0.03	0.01	0.01	0.00
Occasional drinker-no binging	0.08	0.03	**0.50**	**0.56**	0.27	0.21	0.11	0.17	0.04	0.03
Occasional binge drinker	0.09	0.02	0.23	0.32	**0.37**	**0.49**	0.27	0.13	0.04	0.04
Monthly binge drinker	0.03	0.03	0.05	0.04	0.24	0.14	**0.54**	**0.59**	0.14	0.18
Weekly binge drinker	0.00	0.05	0.07	0.00	0.14	0.00	0.30	0.18	**0.48**	**0.77**
**Males**										
**Pre- pandemic (T1)**										
Abstainer	**0.68**	**0.73**	0.18	0.14	0.7	0.08	0.06	0.03	0.01	0.02
Occasional drinker-no binging	0.00	0.00	**0.40**	**0.87**	0.35	0.01	0.22	0.02	0.03	0.10
Occasional binge drinker	0.04	0.00	0.13	0.00	**0.30**	**0.65**	0.49	0.25	0.04	0.01
Monthly binge drinker	0.03	0.08	0.00	0.00	0.11	0.00	**0.62**	**0.61**	0.24	0.31
Weekly binge drinker	0.05	0.00	0.09	0.03	0.68	0.46	0.17	0.48	**0.68**	**0.46**
**Early- pandemic (T2)**										
Abstainer	**0.79**	**0.80**	0.14	0.07	0.04	0.03	0.02	0.02	0.01	0.03
Occasional drinker-no binging	0.07	0.00	**0.47**	**0.49**	0.29	0.07	0.13	0.16	0.03	0.07
Occasional binge drinker	0.10	0.25	0.13	0.00	**0.54**	**0.66**	0.21	0.25	0.02	0.07
Monthly binge drinker	0.05	0.01	0.01	0.00	0.15	0.15	**0.52**	**0.52**	0.28	0.32
Weekly binge drinker	0.05	0.24	0.02	0.03	0.00	0.00	0.17	0.24	**0.76**	**0.71**
